# Role of Vasoactive Hormone-Induced Signal Transduction in Cardiac Hypertrophy and Heart Failure

**DOI:** 10.3390/cells13100856

**Published:** 2024-05-17

**Authors:** Naranjan S. Dhalla, Karina O. Mota, Vijayan Elimban, Anureet K. Shah, Carla M. L. de Vasconcelos, Sukhwinder K. Bhullar

**Affiliations:** 1 Institute of Cardiovascular Sciences, St. Boniface Hospital Albrechtsen Research Centre, Department of Physiology and Pathophysiology, Max Rady College of Medicine, University of Manitoba, Winnipeg, MB R2H 2A6, Canada; velimban@sbrc.ca (V.E.); sbhullar@sbrc.ca (S.K.B.); 2 Department of Physiology, Center of Biological and Health Sciences, Federal University of Sergipe, Sao Cristóvao 49100-000, Brazil; karynamota@academico.ufs.br (K.O.M.); carlamlv@academico.ufs.br (C.M.L.d.V.); 3 Department of Nutrition and Food Science, California State University, Los Angeles, CA 90032-8162, USA; akaur23@calstatela.edu

**Keywords:** vasoactive hormones, cardiac hypertrophy, heart failure, oxidative stress, Ca^2+^-handling abnormalities, extracellular matrix, cardiac dysfunction, ventricular wall tension

## Abstract

Heart failure is the common concluding pathway for a majority of cardiovascular diseases and is associated with cardiac dysfunction. Since heart failure is invariably preceded by adaptive or maladaptive cardiac hypertrophy, several biochemical mechanisms have been proposed to explain the development of cardiac hypertrophy and progression to heart failure. One of these includes the activation of different neuroendocrine systems for elevating the circulating levels of different vasoactive hormones such as catecholamines, angiotensin II, vasopressin, serotonin and endothelins. All these hormones are released in the circulation and stimulate different signal transduction systems by acting on their respective receptors on the cell membrane to promote protein synthesis in cardiomyocytes and induce cardiac hypertrophy. The elevated levels of these vasoactive hormones induce hemodynamic overload, increase ventricular wall tension, increase protein synthesis and the occurrence of cardiac remodeling. In addition, there occurs an increase in proinflammatory cytokines and collagen synthesis for the induction of myocardial fibrosis and the transition of adaptive to maladaptive hypertrophy. The prolonged exposure of the hypertrophied heart to these vasoactive hormones has been reported to result in the oxidation of catecholamines and serotonin via monoamine oxidase as well as the activation of NADPH oxidase via angiotensin II and endothelins to promote oxidative stress. The development of oxidative stress produces subcellular defects, Ca^2+^-handling abnormalities, mitochondrial Ca^2+^-overload and cardiac dysfunction by activating different proteases and depressing cardiac gene expression, in addition to destabilizing the extracellular matrix upon activating some metalloproteinases. These observations support the view that elevated levels of various vasoactive hormones, by producing hemodynamic overload and activating their respective receptor-mediated signal transduction mechanisms, induce cardiac hypertrophy. Furthermore, the occurrence of oxidative stress due to the prolonged exposure of the hypertrophied heart to these hormones plays a critical role in the progression of heart failure.

## 1. Introduction

Heart failure is a major public health problem affecting about 26 million people globally; there are 5.7 million in the United States alone and 670,000 new cases every year [1,2]. This pathological state is associated with cardiac dysfunction, as well as changes in electrical properties and myocardial metabolism, leading to the inability of the heart to pump sufficient blood to meet the oxygen supply and nutrient demands of other organs in the body [3,4]. Heart failure is characterized by shortness of breath, decreased exercise tolerance as well as fluid retention, and when accompanied by arrhythmias, there occurs a high rate of sudden cardiac death. Several cardiovascular diseases such as coronary artery disease, hypertension, aortic valve stenosis, mitral valve regurgitation, inflammatory disease, genetic cardiomyopathy, diabetes and obesity eventually lead to the development of heart failure [5,6,7]. The lifetime risk of developing heart failure is 1 in 5 and the long-term survival is very poor; up to one-third of patients die within the first 12 months and about half of them die within 5 years. The mortality due to heart failure in men is about 60% whereas it is about 45% in women [8,9,10]. Thus, heart failure is a very serious disorder and one of the most common causes of death.

Over the past 60 years, several mechanisms have been proposed to explain the pathogenesis of heart failure [11,12,13,14,15,16]. These include (a) defects in energy production and utilization, (b) increased preload and afterload, (c) elevated levels of vasoactive hormones and altered signal transductions, (d) the development of intracellular Ca^2+^-overload and Ca^2+^-handling abnormalities and (e) cardiac remodeling and subcellular defects. The pathophysiology of cardiac remodeling during the development of heart failure has been reviewed extensively [17,18,19,20,21,22,23,24]. Although all these pathologic mechanisms have been helpful in developing a wide variety of interventions for the therapy of cardiac dysfunction in heart failure, the research based on these issues has not provided sufficient information for distinguishing different types of heart failure such as (a) heart failure with reduced ejection fraction, (b) heart failure with preserved ejection fraction, (c) systolic heart failure and (d) diastolic heart failure [25,26,27,28,29,30]. It may be noted that heart failure with a reduced ejection fraction is caused by myocardial infarction and is usually associated with loss of cardiomyocytes and replacement fibrosis. On the other hand, heart failure with a preserved ejection fraction is seen in patients with chronic hypertension and chronic diabetes and is associated with cardiomyocyte stiffness and interstitial fibrosis. It can be argued that a description of some other distinctive features of heart failure with a preserved ejection fraction versus that with a reduced ejection fraction would enhance the presentation. Since the exact pathophysiological mechanisms with respect to the involvement of vasoactive hormones in heart failure with preserved ejection fraction are not fully understood at present, it would be prudent to focus the discussion on this topic, mainly on the pathogenesis of heart failure with a reduced ejection fraction.

Since heart failure is usually preceded by cardiac hypertrophy, it is of critical importance that events leading to cardiac hypertrophy be understood to define the progression of heart failure. Furthermore, cardiac hypertrophy under different situations at the initial stages serves as an adaptative mechanism and is considered to be compensatory or physiological hypertrophy. However, if the stimulus is not removed, there occurs a transition from physiological cardiac hypertrophy to pathological hypertrophy. The mechanisms for the transition of physiological (adaptive) cardiac hypertrophy to pathological (maladaptive) hypertrophy as well as the characteristics of both these forms of cardiac hypertrophy are reviewed elsewhere [31,32,33,34,35,36,37,38,39,40]. It is emphasized that cardiac hypertrophy, as seen due to exercise, is not always of a pathological nature. Furthermore, the combination of fibrosis and hypertrophy is considered to be a hallmark of pathological hypertrophy as well as cardiac remodeling and dysfunction. It is pointed out there are three types of cardiac hypertrophy that develop to reduce the ventricular wall; these include (a) concentric cardiac hypertrophy, where the muscle mass is increased via the thickening of cardiomyocytes as seen in response to pressure overload; (b) eccentric cardiac hypertrophy, which is associated with an increase in muscle mass via the lengthening of cardiomyocytes as seen in response to volume overload and (c) mixed concentric and eccentric cardiac hypertrophy, where the muscle is increased both via the thickening and lengthening of cardiomyocytes as seen in response to myocardial damage due to myocardial infarction. These structural changes in the size and shape of myocardium in both physiological and pathological forms of cardiac hypertrophy are generally indicated as cardiac remodeling and it is the pathological (adverse) cardiac hypertrophy that results in the development of heart failure.

**Figure 1:**
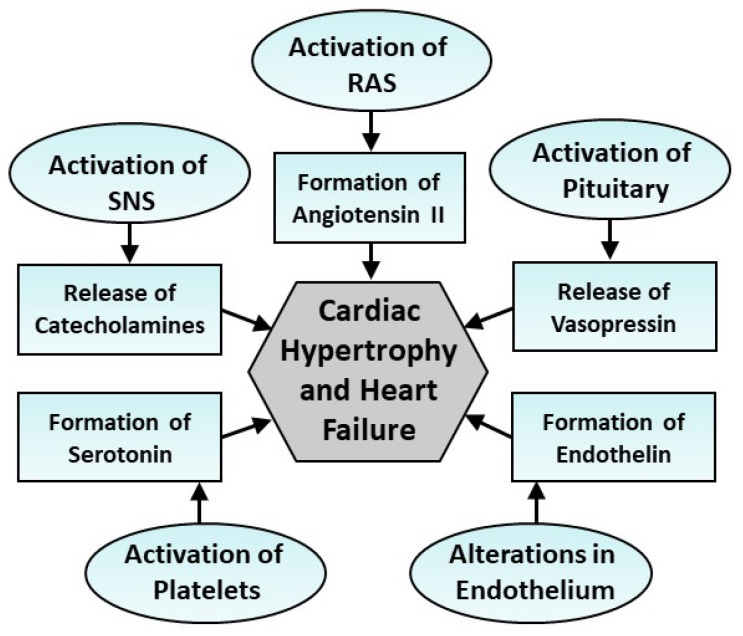
Some endocrine systems involved in the development of cardiac hypertrophy and heart failure through the release of different vasoactive hormones. SNS—sympathetic nervous system; RAS—renin–angiotensin system.

Several neuroendocrine systems and pathological factors are not only inter-related with each other but are also intimately involved in the development of cardiac hypertrophy as well as the progression of heart failure [18,20,24,35,41,42,43,44,45,46,47]. Some of the endocrine systems are shown in Figure 1. It may be noted that myocardial injury due to myocardial infarction is known to result in activations of the sympathetic nervous system (SNS), posterior pituitary, endothelium and platelets, which result in the release of different vasoactive hormones such as norepinephrine, vasopressin, endothelins and serotonin, respectively, whereas the activation of the renin–angiotensin system (RAS) promotes the formation of angiotensin II upon the release of renin from the kidney. These vasoactive hormones have also been shown to induce the development of pathological stimuli, including pressure overload, volume overload, inflammation, increased ventricular wall tension and other abnormalities during the development of cardiac hypertrophy and heart failure. However, only scattered information is available regarding mechanisms for the transition of cardiac hypertrophy to heart failure.

Although plasma levels of several vasoactive neurohumorals and growth factors are elevated in cardiac hypertrophy and heart failure [41,42,43,44,45,46,47,48,49], mechanisms of their release as well as formation seem to depend upon the type and stage of pathological stimulus. For example, a depression in cardiac output and a decrease in blood pressure due to myocardial infarction, cardiomyopathies and inflammatory diseases stimulate the SNS and peripheral RAS for the release of catecholamines (norepinephrine from the sympathetic nerve endings and epinephrine from the adrenal medulla) and promote the release of renin from the kidney for the formation of Ang II, respectively. Activations of the posterior pituitary for the release of vasopressin and the hypothalamic center for the release of different growth factors are also considered to be due to a decrease in blood pressure. On the other hand, the release of vasoactive hormones and growth factors under conditions of pressure overload and volume overload is considered to occur as a consequence of increased ventricular pressure stress and the activation of afferent nerve fibers connected to different centers in the brain. In addition, increased ventricular wall stress due to hemodynamic overload can be seen to affect vascular endothelium in the heart to release endothelins, mast cells and serotonergic nerve fibers to release serotonin, sympathetic nerve endings in the ventricle to release norepinephrine and endogenous RAS to release angiotensin II. The activation and aggregation of platelets by circulating norepinephrine and angiotensin II are the major sources of elevated levels of serotonin. These several vasoactive hormones and growth factors are considered to play an important role in the maintenance of hemodynamic homeostasis, the development of cardiac hypertrophy and the progression of heart failure.

It is commonly held that prolonged exposure of the hypertrophied heart to high levels of circulating vasoactive hormones or different pathological stimuli results in the decompensation of the hypertrophied heart for the progression to heart failure. This article, therefore, deals with a comprehensive discussion of signal transduction mechanisms for the development of cardiac hypertrophy as well as an updated description of events that result in the progression of cardiac hypertrophy to heart failure. Although elevated levels of several other hormones such as aldosterone, thyroid hormone, arterial natriuretic peptide (ANP), brain natriuretic peptide (BNP), insulin/insulin-like growth factor and other growth factors and NO are observed in heart failure [48,49,50,51,52,53,54,55,56,57], the discussion on these aspects is not included in this review. In fact, this article is intended to focus on the discussion of some vasoactive hormones, which are known to produce hemodynamic overload and increase ventricular wall tension. Since vasoactive hormones are also known to release different proinflammatory cytokines and produce myocardial fibrosis, their participation in the transition from adaptive cardiac hypertrophy to maladaptive hypertrophy will be discussed. Furthermore, in view of the critical role of vasoactive hormones in inducing oxidative stress and Ca^2+^-handling abnormalities in cardiomyocytes [58,59,60,61,62,63,64,65], it is planned to highlight their involvement in the development of cardiac dysfunction and heart failure.

## 2. Role of Catecholamines in Cardiac Hypertrophy and Heart Failure

It is now well known that circulating levels of both norepinephrine and epinephrine are increased during the development of heart disease under a wide variety of stressful conditions [66,67]. In the initial stages, elevated levels of plasma catecholamines produce adaptive changes in the heart for maintaining cardiac function; however, at later stages, these hormones result in cardiac dysfunction and cardiomyopathy. Catecholamines have been demonstrated to increase cardiac function and induce cardiac hypertrophy via β-adrenoceptors, activating Gs-protein–adenylyl cyclase complex and promoting the formation of cyclic AMP [17,68,69,70,71]. The increased concentration of cyclic AMP activates protein kinase A (PKA) and phosphorylates various intracellular sites to increase Ca^2+^-movements and protein synthesis in cardiomyocytes. In fact, catecholamines have been reported to stimulate subcellular Ca^2+^-transport, cardiac gene expression and protein synthesis for the induction of adaptive changes in the heart [72,73,74]. These alterations through elevated levels of circulating catecholamines in cardiac hypertrophy were attenuated through the β-adrenoceptor blockade as well as Ca^2+^-antagonists [75]. Furthermore, the inhibition of extracellular signal-regulated kinases (ERK 1/2) was found to abolish the catecholamine-induced cardiac gene expression [74]. It appears that the β-adrenoceptors-PKA-ERK1/2-associated signal transduction system may be involved in the initial hypertrophic response as well as in augmenting cardiac function via catecholamines.

The increase in the cardiac contractile activity and hypertrophic growth action of catecholamines under physiological conditions is mainly modulated through β-adrenoceptor-associated mechanisms [17,76]; however, α-adrenoceptor-associated signal transduction is considered to become more apparent for initiating the progression of cardiac hypertrophy under pathological situations [77]. The activation of α-adrenoceptors via norepinephrine has been shown to stimulate phospholipase C (PLC), which is coupled with G_q_-proteins, and results in the production of 1,2 diacylglycerol (DAG) and inositol -1, 4, -5 triphosphate (IP3), the activation of protein kinase C (PKC) and mitogen-activated protein kinase (MAP kinase, ERK1/2). The activation of this signaling pathway is associated with the release of intracellular Ca^2+^ and the induction of hypertrophic response in cardiomyocytes [78]. The development of cardiac hypertrophy in spontaneously hypertensive rats, cardiomyopathic hamsters and volume-overloaded rats has been shown to be associated with the activation of PLC [79,80,81]. Norepinephrine-induced cardiac hypertrophy, gene expression and protein synthesis were attenuated via U73122, an inhibitor of PLC, as well as prazosin, an α-adrenergic receptor blocker [82]. A depression in norepinephrine-stimulated gene expression and protein synthesis in cardiomyocytes via bisindolylmaleimide -1, a PKC inhibitor, and PL98059, an ERK1/2 inhibitor, indicated that PKC-ERK1/2 may be involved in the PLC-associated signal transduction pathway. It is pointed out that the stimulation of protein synthesis in cardiomyocytes has also been reported to occur through phosphatidic acid, a product of phospholipase D activation, for the development of cardiac hypertrophy [83,84]. Thus, phospholipid-mediated signal transduction upon the activation of α-adrenoceptors may represent an important mechanism for the occurrence of cardiac hypertrophy due to catecholamines.

**Figure 2:**
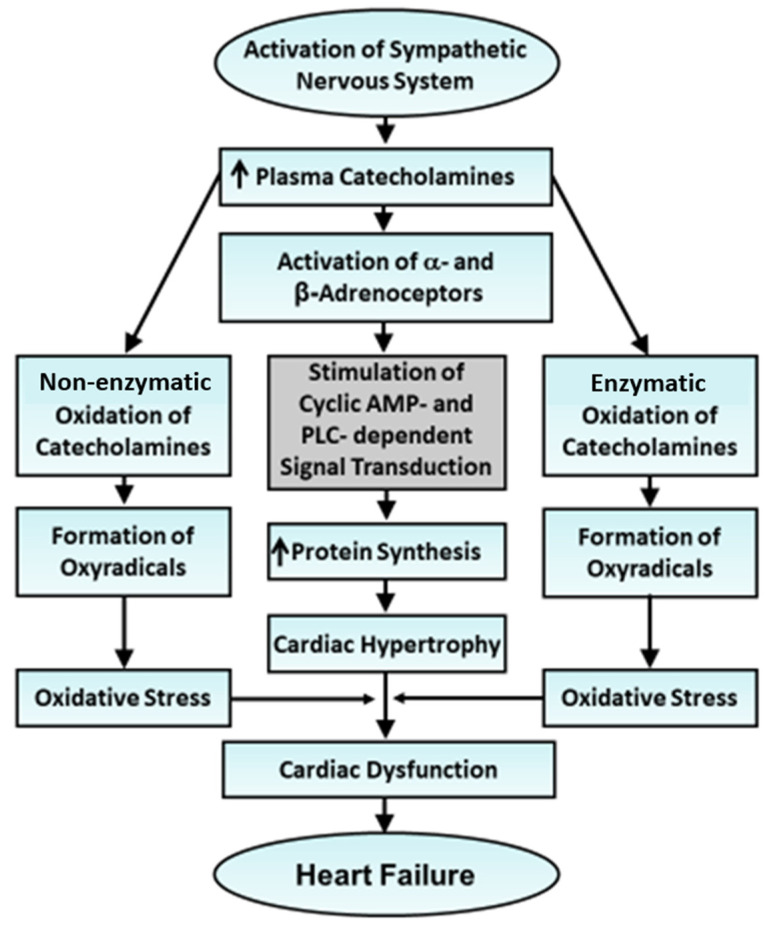
Role of the sympathetic nervous system in the development of cardiac hypertrophy and progression of heart failure. PLC—phospholipase C. Low concentrations of plasma catecholamines upon the activation of adrenoceptors induce cardiac hypertrophy, whereas high concentrations upon oxidation for a prolonged period result in heart failure.

It needs to be emphasized that the actions of catecholamines at the initial stages are beneficial for maintaining cardiac function, but their delayed effects are deleterious [17,66,67]. A schematic representation of events depicting the involvement of both β-adrenoceptors and α-adrenoceptors in causing cardiac hypertrophy and the role of oxidative stress in the occurrence of heart failure at the later stages of catecholamine action is shown in Figure 2. It may be noted that β-adrenoceptor signal transduction is downregulated due to high levels of circulating catecholamines for a prolonged period and, thus, there occurs a depression in Ca^2+^-transport in cardiomyocytes, leading to the development of cardiac dysfunction [17,72,85]. Such a decrease in subcellular Ca^2+^-transport via high levels of plasma catecholamines has been shown to be a consequence of the occurrence of oxidative stress [86]. It is also pointed out that oxyradicals are generated during the oxidation of catecholamines through both non-enzymatic and enzymatic mechanisms and under conditions where the endogenous antioxidant pool becomes saturated or depressed, these oxyradicals lead to the development of oxidative stress [87,88]. Catecholamines have also been demonstrated to accumulate in cardiomyocytes, become oxidized through mitochondrial monoamine oxidase and generate oxyradicals [89]. Furthermore, the cardiotoxic effects of high levels of catecholamines are prevented via antioxidants such as vitamin E, vitamin A, vitamin C, N-acetyl L-cysteine and sulfur-containing amino acids [90,91,92]. It may be noted that in spite of several epidemiological and experimental studies showing the beneficial effects of different vitamins in attenuating cardiovascular disorders, several clinical investigations to determine the therapeutic effects of vitamins such as E or C have shown inconclusive and inconsistent results [91]. Accordingly, it was suggested that antioxidant vitamins may be involved in the prevention rather than the therapy of cardiovascular disease [91]. Nonetheless, different adrenoceptor antagonists have been shown to exert beneficial effects in heart failure not only by exerting antioxidant effects or attenuating cardiac hypertrophy upon blocking α- or β-adrenoceptors but also by lowering the elevated levels of plasma catecholamines upon acting on the sympathetic nerve terminals [93,94,95]. These observations suggest the involvement of oxidative stress in cardiac dysfunction during the development of catecholamine-induced cardiomyopathy.

## 3. Role of Angiotensin II in Cardiac Hypertrophy and Heart Failure

Over the past six decades, extensive research has been carried out to understand the involvement of angiotensin II (Ang II) in the development of hypertension, cardiac hypertrophy and heart failure [96,97,98,99,100,101,102,103,104]. Ang II is a multifunctional hormone, which is formed in both peripheral (circulating) and local (tissue) RAS. The generation of Ang II in the peripheral RAS is associated with the release of renin from the kidneys via a reduction in blood flow, the formation of Ang I from angiotensinogen in the liver by renin and the conversion of Ang I to Ang II in the lung with the angiotensin-converting enzyme (ACE). Although all components of the RAS are expressed in various organs, ACE is mainly localized on fibroblasts as well as the endothelium; chymase is also involved in the conversion of Ang I to Ang II in the heart. Thus, under a wide variety of pathophysiological conditions, the circulating levels of Ang II are elevated upon the activation of the peripheral RAS via reduced blood flow to the kidneys, whereas the activation of the cardiac RAS is stimulated by increased ventricular wall tension to maintain hemodynamic homeostasis. Several excellent reviews concerning the pathophysiological implications of the activated RAS as well as the mechanisms of Ang II action on the cardiovascular system are available in the literature [105,106,107,108,109,110]. Although the exact time course for the activation of the RAS due to any pathophysiological stimulus still remains to be established, it seems that the activation of the RAS may lag behind that of the SNS because of the time required for the synthesis of Ang II from angiotensinogen in the peripheral RAS as well as the development of the ventricular wall tension for the release of Ang II from the cardiac RAS.

The elevated levels of Ang II not only produce vasoconstriction, cardiac hypertrophy and heart failure but are also involved in release of catecholamines from the sympathetic nerve terminals and the adrenal medulla for raising blood pressure, the release of aldosterone from the adrenal cortex for salt and fluid retention and the release of autocrine factors such as transforming growth factors (TGF-β) and interleukin (IL-6) for inflammatory responses [100,101,102,104,107]. The cardiovascular effects of Ang II are mediated by two types of receptors, namely AT_1_R and AT_2_R. The interaction of Ang II with AT_1_R induces prohypertensive, prohypertrophic and proinflammatory actions, whereas the interaction of Ang II with AT_2_R has been shown to produce antihypertensive, antihypertrophic and anti-inflammatory effects [102,104,111,112,113,114]. Both Ang I and Ang II are metabolized to Ang (1–9) and Ang (1–7) with ACE2, the homologue of ACE, which is known to activate MAS receptors (MASR) and produce antihypertensive, anti-inflammatory and antihypertrophic actions [115,116,117]. Since the effects of AT_1_R activation are antagonized by the effects of AT_2_R activation and MASR activation, an imbalance between the adverse actions of AT_1_R activation and the beneficial effects of AT_2_R activation as well as MASR activation has been suggested to determine the acceleration and progression of heart disease [104,118,119]. Although AT_1_R, AT_2_R and MASR are G_q-_ protein-coupled receptors [120,121], the sequence of their activations during the development of cardiac hypertrophy and heart failure has not yet been established.

The activation of the peripheral RAS has been shown to increase the level of plasma Ang II rapidly and stimulate AT_1_R in vascular smooth muscles to elevate blood pressure and produce a hypertrophic response [102,104]. The elevated blood pressure is considered to increase afterload on the heart and, thus, increase the left ventricular wall tension to activate the local RAS and release Ang II. Thus, Ang II from both peripheral and local sources activates AT_1_R in cardiomyocytes to stimulate myocardial metabolism and cardiac function in addition to inducing signal transduction for the process of cardiac hypertrophy. In the early stages, the activation of AT_1_R is associated with the incorporation of different amino acids and the stimulation of a signal transduction mechanism for the synthesis of proteins and the development of adaptive cardiac hypertrophy [102,104,122,123]. The Ang II- AT_1_R hypertrophic signal transduction includes the activation of sarcolemmal PLC, resulting in (i) the formation of DAG, the stimulation of PKC and the activation of MAP kinase and (ii) the formation of IP_3_, the release of Ca^2+^ from the sarcoplasmic reticulum and the activation of Ca^2+^-calmodulin kinase for the occurrence of cardiac hypertrophy. It is noteworthy that AT_1_R is coupled with NADPH oxidase (NOX)-2 in the sarcolemmal membrane, whereas the production of small amounts of oxyradicals upon activating AT_1_R is considered to change the redox status of cardiomyocytes and promote the hypertrophic signal transduction pathway for the induction of adaptive cardiac hypertrophy [102,104].

Since Ang II is known to activate AT_2_R and its metabolite, Ang (1–7) activates MASR to produce antihypertrophic responses for limiting the development of cardiac hypertrophy induced through AT_1_R activation [102,104]; the net growth of myocardium due to Ang II is considered to be a balance between the effects of AT_1_R activation and AT_2_R as well as MASR activations. Several immediate early genes including c-fos, c-jun and c-myc are also induced through the activation of AT_1_R for the development of cardiac hypertrophy [122,123,124]. The activation of AT_1_R in the adrenal cortex by Ang II has also been documented to release aldosterone and promote cardiac hypertrophy as a consequence of increasing the preload on the heart due to its sodium retention and fluid-accumulating effects [103,104,125]. Thus, the development of adaptive cardiac hypertrophy via Ang II not only involves the AT_1_R-mediated signal transduction pathway but is also a consequence of increased ventricular wall tension due to hemodynamic overload. It should also be noted that Ang II has been shown to release proinflammatory cytokines such as interleukin (IL)-6, IL-1β and tumor necrosis factor (TNF)-α and anti-inflammatory cytokines including IL-10 and transforming growth factor (TGF)-β from macrophages and neutrophils [13,104,126,127]. These proinflammatory cytokines are known to promote the accumulation of collagen in the extracellular matrix and fibrosis in cardiomyocytes. The progressive Ang-II-induced activation of different isoforms of NOX has also been reported to exhaust the antioxidant reserve and increase the concentration of oxyradicals [102,128,129,130,131,132,133,134] in cardiomyocytes. Such an effect of Ang II has been shown to be associated with the occurrence of apoptosis and fibrosis. Thus, it appears that both myocardial inflammation and high levels of oxyradicals may be involved in the transition of adaptive cardiac hypertrophy to maladaptive cardiac hypertrophy due to elevated levels of Ang II.

**Figure 3:**
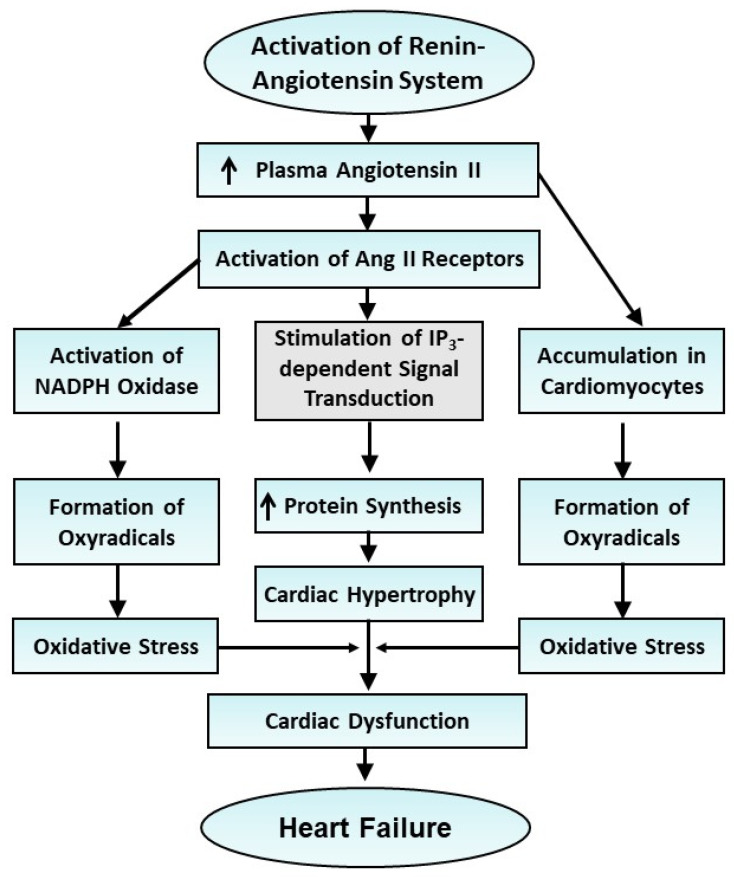
Role of the renin–angiotensin system in the development of cardiac hypertrophy and the progression of heart failure. Ang II—angiotensin II. Elevated plasma levels of angiotensin upon the activation of Ang II receptors induce cardiac hypertrophy, whereas the prolonged activation of NADPH oxidase results in heart failure.

It is now becoming clear that acute exposure to Ang II is associated with the development of adaptive cardiac hypertrophy in which cardiac function is either unaltered or augmented, whereas prolonged exposure of the heart to Ang II results in the transition of adaptive cardiac hypertrophy to maladaptive cardiac hypertrophy, in which cardiac function is depressed; thereafter, a progression to a major health hazard occurs, namely heart failure. A simplified schematic representation of some major events occurring during the development of Ang II-induced cardiac hypertrophy and heart failure is shown in Figure 3. Although the exact mechanisms associated with the progression of heart failure subsequent to various pathological conditions are of a complex nature and not fully understood, the involvement of Ang II in this process is evident from the fact that various blockers of the RAS and AT_1_R antagonists are well known to produce significant beneficial effects [103,104]. Furthermore, a progressive increase in the degree of oxidative stress has been claimed to be associated with the progression of heart failure [102,128,129,130,131,132,133,134]. There are four major mechanisms that have been identified in the production of oxidative stress due to prolonged exposure to Ang II [101,102,103,104,105,106,107,108,109,110,111,112,113,114,115,116,117,118,119,120,121,122,123,124,125,126,127,128,129,130,131,132,133,134,135,136,137,138,139,140,141,142,143,144,145,146,147,148,149,150,151,152,153,154,155,156,157,158,159,160,161,162,163,164]. These include (i) the activation of NOX-2 and NOX-4 during the hypertrophic process; (ii) the exhaustion of antioxidant reserve due to the continued activation of AT_1_R and the depressed activity of nuclear factor erythroid-2 elated factor 2; (iii) the stimulation of NOX-4 in mitochondria upon the entry of Ang II in cardiomyocytes and (iv) the induction of functional hypoxia in the hypertrophied myocardium due to the inadequate development of capillaries in comparison to cardiomyocytes growth. The excessive development of oxidative stress in cardiomyocytes has been reported to adversely affect the function of different subcellular organelles and result in Ca^2+^-handling abnormalities, metabolic alterations, changes in cardiac gene expression and the impairment of cardiac performance [58,59,60,61,62,101,102,103,104,134]. Although the role of cardiac inflammation cannot be ruled out, the evidence available in the literature strongly supports the view that oxidative stress plays a crucial role in the progression of heart failure due to prolonged exposure to Ang II.

## 4. Role of Serotonin in Cardiac Hypertrophy and Heart Failure

Serotonin (5-hydroxytryptamine; 5-HT) is a monoamine, which is present in platelets, mast cells and sympathetic nerve terminals in the heart [135,136]. The release of this hormone upon the aggregation of platelets as well as the degranulation of mast cells has been shown to produce vasoconstriction, smooth muscle cell proliferation, coronary spasm, tachycardia, inotropic effect, cardiac hypertrophy and fibrosis [137,138,139,140,141]. Although there are several families of serotonin receptors present in the cardiovascular system, the vasoconstriction and hypertrophic effects of this hormone are mainly mediated via 5-HT_2A_ and 5-HT_2B_ receptors [142,143,144]. The activation of 5-HT_2A_ receptors, which are coupled with PLC through G_αq_ proteins, has been shown to stimulate PKC due to the formation of DAG and induce a hypertrophic response involving MAP kinase [145]. Furthermore, the activation of these receptors with serotonin has been demonstrated to accumulate IP_3_ upon the hydrolysis of phosphoinositide for releasing Ca^2+^ from the intracellular pool [146,147,148]. Although the stimulation of both SNS and RAS is also known to activate platelets and release serotonin, plasma levels of serotonin are increased due to ischemia-reperfusion, atherosclerosis, coronary artery disease and heart failure [149,150,151,152,153,154]. Accordingly, the serotonin-5-HT_2A_ signal pathway is considered to regulate cardiovascular function in both health and disease [149,150,151,152,153,154,155,156].

Several antiplatelet agents such as aspirin, clopidogrel and cilostazol, either alone or in combination, have been reported to produce beneficial effects in diverse cardiovascular diseases including pulmonary hypertension [157,158], coronary artery abnormalities [159,160,161,162,163] and ventricular arrhythmias and atrial fibrillation [155,156,164]. Furthermore, the 5-HT_2A_ receptor antagonist, ketanserin, has been shown to improve hemodynamic and neurohumoral alterations in patients with heart failure [165,166]. Sarpogrelate, another 5-HT_2A_ receptor antagonist, was also demonstrated to mitigate cardiac remodeling as well as subcellular remodeling in heart failure due to myocardial infarction [167,168]. Sarpogrelate has been reported to suppress Ang II-, endothelin-1- or phenylephrine-induced cardiac hypertrophy in cultured cardiomyocytes in addition to attenuating systolic dysfunction in mice subjected to transverse aortic constriction [169]. Since sarpogrelate was found to inhibit the effects of different stimuli other than serotonin, it has been suggested that this agent may affect some hypertrophic signaling other than that associated with 5-HT_2A_ activation [169]. Nonetheless, these observations support the view that serotonin is involved in the pathogenesis of cardiovascular abnormalities, and it appears that various antiplatelet agents and 5-HT_2A_ antagonists may not be specific for acting on the same site in the hypertrophic signal transduction pathway for serotonin.

Serotonin not only exerts vasoconstriction and raises blood pressure but is also known to act as a growth factor, stimulating mitogenesis and migration of arterial smooth muscle cells [143,144,170,171]. It produces cardiostimulatory effects [172,173] and is involved in the development of cardiac hypertrophy as well as heart failure [151,155,156]. The plasma levels of serotonin are correlated with the progression of heart failure involving CaMK II/HDAC 4 signal transduction [150,174,175,176,177]. The activation of 5-HT_2A_ receptors for the induction of cardiac hypertrophy was observed to be associated with the ERK ½-GATA4 signal pathway [169]. Elevated levels of plasma serotonin were also reported in patients with diastolic heart failure and ischemic heart disease and were formed to activate different receptors such as 5-HT_2B_ and 5-HT_4_ [178,179,180,181,182,183,184]. Serotonin has also been shown to play an important role in regulating cardiac development and function through the involvement of HT_2B_ receptors, and in fact, the overexpression of 5-HT_2B_ receptors has been demonstrated to induce cardiac hypertrophy [185,186]. The interleukin-18-induced cardiac hypertrophy was inhibited via pretreatment with the 5-HT_2B_ receptor antagonist, SB215505, as well as siRNA for the 5-HT_2B_ receptor [187]. It may also be noted that aspirin, an antiplatelet agent, has been reported to attenuate the right ventricular hypertrophy due to pulmonary hypertension [158], whereas another antiplatelet agent, cilostazol, was shown to depress myocardial infarction-induced right ventricular hypertrophy [167,168]. Thus, it appears that the involvement of serotonin in the induction of cardiac hypertrophy and heart failure due to different types of pathological stimuli may be associated with different types of serotonin receptors as well as signal transduction pathways.

**Figure 4:**
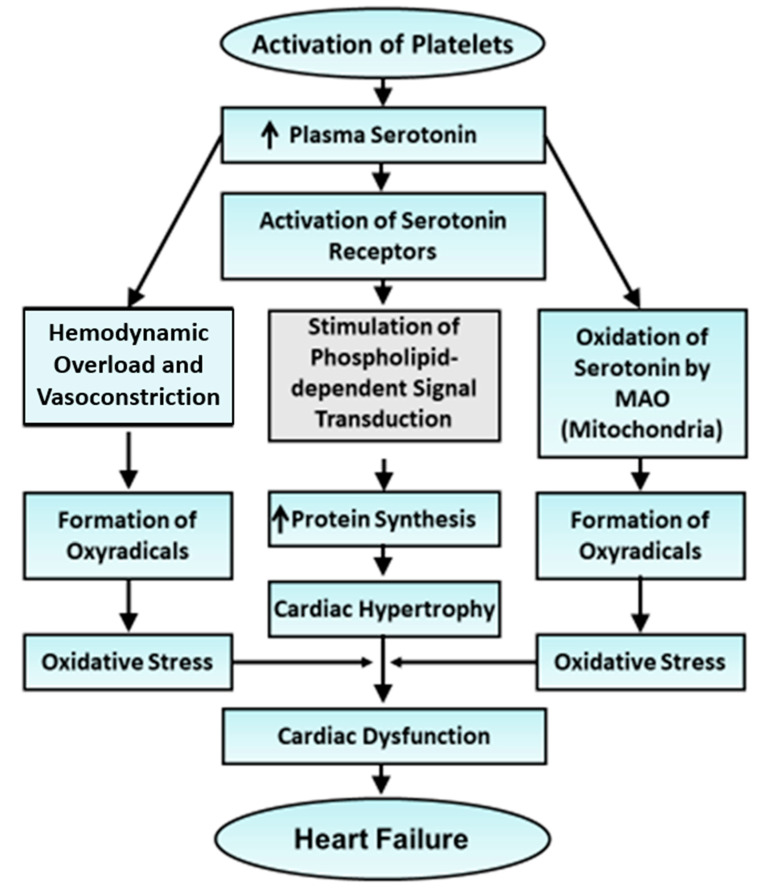
Role of the activation of platelets in the development of cardiac hypertrophy and progression of heart failure. MAO—monoamine oxidase. Cardiac hypertrophy is induced by the activation of serotonin receptors, whereas its transition to heart failure occurs due to the oxidation of serotonin by MAO.

In view of the participation of platelets as a major source for the release of serotonin during the development of cardiac hypertrophy and heart failure, a graphic presentation of signal transduction events associated with pathological situations is given in Figure 4. Elevated levels of serotonin upon activating its receptors and signal transduction pathway promote protein synthesis and induce cardiac hypertrophy. Serotonin-induced vasoconstriction and increased blood pressure can be seen to increase hemodynamic overload on the heart and, thus, would also promote the occurrence of cardiac hypertrophy. Since monoamine oxidase-A (mainly present in mitochondria) is involved in the degradation of serotonin and the production of oxyradicals and H_2_O_2_ [188], it is suggested that serotonin will not only change the redox status of cardiomyocytes for promoting cardiac hypertrophy upon producing a small amount of oxyradicals at initial stages but will also produce oxidative stress, intracellular Ca^2+^-overload, apoptosis and necrosis for the induction of cardiac dysfunction and heart failure [89,188,189,190].

## 5. Role of Endothelin-1 in Cardiac Hypertrophy and Heart Failure

Following the discovery of endothelin in 1988 [191] and the identification of endothelin-1 as the most potent vasoconstrictor [192,193], extensive research has been carried out to understand the role of endothelin-1 in cardiovascular health and disease. Several excellent reviews in the area of endothelin-1 molecular biology, pathophysiology and pharmacotherapy have appeared in the literature [194,195,196,197,198,199,200]. This hormone is produced mainly in the vascular endothelium and is known to increase blood pressure, exert a positive inotropic effect and produce cardiac hypertrophy. In addition, endothelin-1 influences salt and water retention homeostasis due to its interactions with angiotensin II, aldosterone and vasopressin [195]. This hormone is released from the endothelium through hemodynamic shear stress in the ventricle as well as through hypoxia, vasoactive hormones, growth factors and inflammatory cytokines [196]. Low concentrations of endothelin-1 are considered to maintain cardiovascular homeostasis, whereas the excessive production of this vasoactive hormone has been demonstrated to result in hypertension, cardiac hypertrophy and heart failure [195,196]. The cardiovascular effects of endothelin-1 are mediated by two types of receptors, namely ET_A_ and ET_B_ [201]. While the activation of ET_A_ is associated with vascular constriction and cell proliferation as well as myocardial cell growth and cardiac hypertrophy, the activation of ET_B_ has been shown to produce vasodilatory and antiproliferative effects [201]. Thus, the net effect of endothelin-1 on the cardiovascular system seems to be dependent upon the activity ratio of ET_A_/ET_B_.

ET_A_ receptors are present on both vascular smooth muscle cells and cardiomyocytes, whereas ET_B_ receptors are present on endothelial cells [196,202,203]. Endothelin-1 has been shown to produce smooth muscle contraction by activating ET_A_ receptors, whereas it promotes the production of NO in endothelial cells upon the activation of ET_B_ receptors [204,205]. Furthermore, endothelin-1 has been observed to increase contractile force in the heart by activating ET_A_ receptors [206]. Both ET_A_ and ET_B_ receptors are coupled to PLC through G_q_-proteins [207,208]. The activation of ET_A_ in smooth muscle cells and cardiomyocytes results in a hypertrophic response involving PLC-PKC-MAP kinase-mediated signal transduction mechanisms [194,199]. The activation of PLC has also been shown to increase the intracellular concentration of Ca^2+^ for the occurrence of vasoconstriction and cardiostimulation as well as apoptosis [194,195]. In addition, the activation of ET_A_ receptors through endothelin-1 is associated with the stimulation of phosphoinositide 3-kinase and protein kinase Akt (or protein kinase B) for promoting protein synthesis and protecting against the development of apoptosis [194,209,210]. The mitogenic effects of endothelin-1 for the induction of smooth muscle cell proliferation and cardiac growth are associated with the induction of several proto-oncogenes such as c-fos, c-jun and c-myc [194,199].

**Figure 5:**
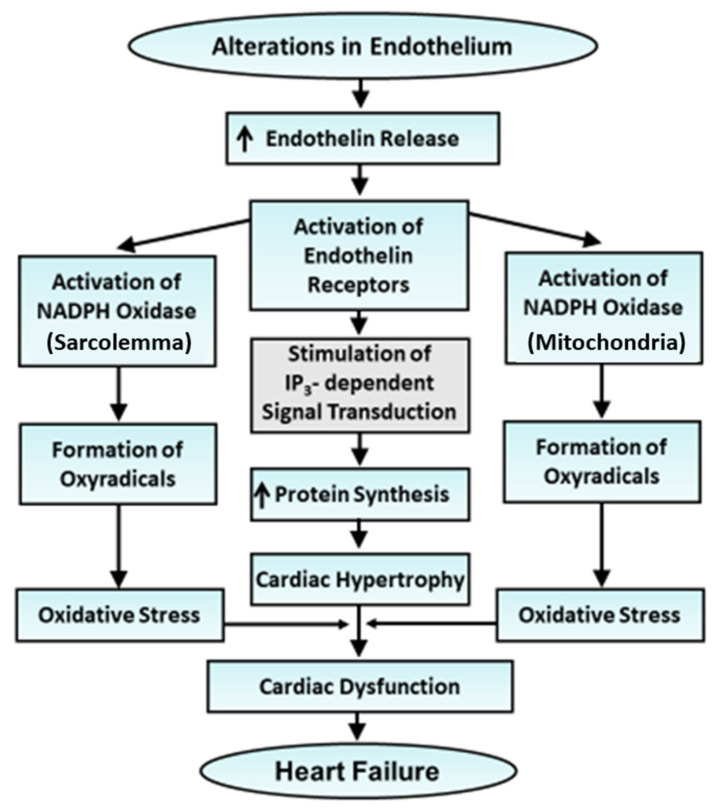
Role of alterations in endothelium in the development of cardiac hypertrophy and progression of heart failure. Cardiac hypertrophy is induced via the activation of endothelin receptors, whereas its transition to heart failure occurs due to the activation of NADPH oxidase.

There is a growing body of evidence to indicate that endothelin-1 is involved in the pathogenesis of hypertension, cardiac hypertrophy and heart failure [194,195,196,199,211,212,213,214,215,216]. The increase in blood pressure due to endothelin-1 can be seen to increase the left ventricular pressure and induce cardiac hypertrophy. It may also be noted that the induction of pulmonary hypertension as a consequence of elevated levels of endothelin-1 would result in hypertrophy of the right ventricle leading to right heart failure [215,216]. The role of endothelin-1 in the development of pulmonary hypertension and right heart hypertrophy is further substantiated by the fact that several ET_A_ antagonists such as bosentan, macitentan and ambrisentan have been shown to produce beneficial effects in patients with pulmonary hypertension [194,198,217,218]. In addition to hemodynamic overload, endothelin-1 induces cardiac hypertrophy upon binding with ET_A_ receptors and the stimulation of the PLC-mediated signal transduction pathway [218,219,220,221]. It is also pointed out that plasma levels of endothelin-1 have been reported to increase in heart failure due to different pathological situations [222,223,224,225,226,227]. In fact, there occurs a positive correlation between plasma levels of endothelin-1 and the degree of cardiac dysfunction in heart failure [228]. Furthermore, endothelin-1 has been demonstrated to activate NOX for the generation of oxidative stress via the involvement of the ET_A_-proline-rich tyrosine kinase-2 and Rac 1 pathway [229] and, thus, can be seen to induce heart failure. Treatments with ET_A_ antagonists such as bosentan and BQ-123 have been shown to improve the cardiac function and survival of heart failure subjects [230]. Several endothelin-1 receptor blockers [197,231] and salidroside, an antioxidant [232], have also been demonstrated to inhibit adverse cardiac remodeling in heart failure. A schematic representation of events for the development of cardiac hypertrophy and heart failure due to endothelin-1 is shown in Figure 5.

## 6. Role of Vasopressin in Cardiac Hypertrophy and Heart Failure

Vasopressin is a nonapeptide hormone with a six-member disulfide ring and a three-member tail with a terminal carboxyl group [233,234]. This hormone is produced in supraoptic and paraventricular nuclei of the hypothalamus and stored in the posterior pituitary. Vasopressin is secreted in response to the activation of both osmotic and non-osmotic receptors for maintaining body fluid homeostasis and peripheral vascular resistance under several pathological conditions [47,197,235,236,237]. The osmotic secretion of this hormone is regulated by osmoreceptors in the hypothalamus, which sense small changes in plasma osmolarity due to alterations in sodium concentrations and results in the retention of water rather than sodium [238,239]. On the other hand, the non-osmotic release of vasopressin is controlled by baroreceptors in the left atrium, aortic arch and carotid sinus in response to atrial underfilling due to a decrease in cardiac output or peripheral vascular resistance [240]. The synthesis of vasopressin has also been reported to occur in the heart in response to pressure overload [241] but the significance of the hormone action from this source is not clear except that it may exert some local or systemic effect. The activation of the SNS is considered to promote the production of vasopressin [240], whereas Ang II has been shown to affect its release [242].

**Figure 6:**
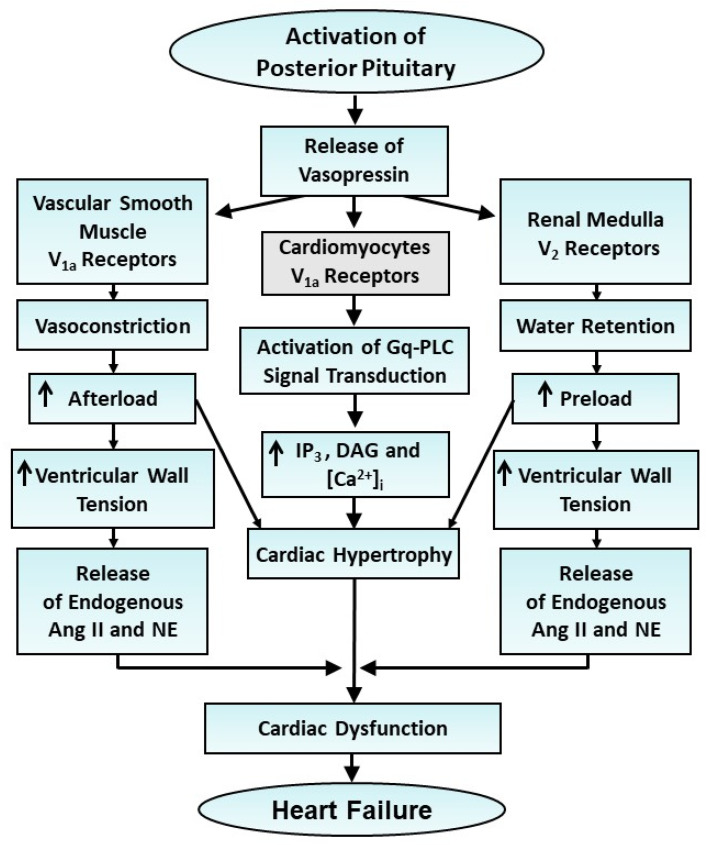
Role of the elevated levels of vasopressin in cardiac hypertrophy as well as vasoconstriction and water retention for the development of increased afterload and preload and subsequent cardiac dysfunction as well as heart failure.

There are two major G-protein-coupled vasopressin receptors, namely the V_1a_ receptor and the V_2_ receptor, which mediate the cardiovascular responses of this hormone in the body [243]. The activation of V_1a_ receptors has been demonstrated to increase contractile force in the heart [244] and produce cardiac hypertrophy [245]. The increase in blood pressure by vasopressin due to its action on vascular smooth muscle cells can be seen to increase the afterload on the heart and promote the occurrence of cardiac hypertrophy. Vasopressin has been reported to cause cardiac growth by promoting protein synthesis in neonatal and adult cardiomyocytes [245,246]. It is pointed out that the interaction of V_1a_ receptors with vasopressin results in the activation of PLC-mediated signal transduction, involving the stimulation of PKC and MAP kinase as well as the increase in the concentration of Ca^2+^ for augmenting protein synthesis in cardiomyocytes and smooth muscle myocytes [247,248]. On the other hand, the activation of V_2_ receptors, which are mainly located on the basolateral membrane in the renal medulla, leads to water retention in the body [248,249]. This antidiuretic hormone has also been shown to stimulate adenylyl cyclase, increase the intracellular concentration of cyclic AMP and activate protein kinase A for increasing the rate of insertion of water channel-containing vesicles into apical membrane [250]. Such an action of V_2_ receptor activation increases water permeability [249] for increasing fluid accumulation in the body, which is known to produce the preload on the heart. Thus, vasopressin is considered to increase both the afterload and preload on the heart by activating the V_1a_ receptors and V_2_ receptors, respectively. This hemodynamic overload on the hypertrophied heart would increase the ventricular wall tension and release endogenous Ang II and norepinephrine, which are known to promote the occurrence of oxidative stress and induce heart failure. Accordingly, it appears that the transition of vasopressin-induced cardiac hypertrophy to heart failure may also occur due to the development of oxidative stress as a consequence of both Ang II and norepinephrine released from endogenous RAS and sympathetic nerve endings in the heart. A schematic representation of events for the induction of cardiac hypertrophy and heart failure due to vasopressin is given in Figure 6.

It has been reported that the plasma levels of vasopressin are elevated during the development of heart failure [251,252,253]. While the activation of the V_1a_ receptors via vasopressin results in the development of vasoconstriction, hypertension, cardiac hypertrophy and heart failure, the activation of the V_2_ receptors is associated with fluid retention, leading to the development of volume overload, venous congestion, edema and lung congestion [254,255,256,257,258,259]. Vasopressin not only causes water retention but also results in kidney dysfunction in heart failure patients. These abnormalities are associated with the occurrence of hyponatremia, which may limit the use of several agents such as diuretics for the management of heart failure [260,261,262,263]. Nonetheless, various therapies based on the antagonist effects of different agents on both the V_1a_ receptor and V_2_ receptors have been developed for the treatment of heart failure [197]. Since these vasopressin blockers improve cardiac function and reduce cardiac hypertrophy, it can be argued that vasopressin plays an important role in the pathogenesis of cardiac hypertrophy and heart failure.

## 7. Perspective and Concluding Remarks

Various cardiovascular diseases such as myocardial infarction, hypertension, diabetes, aortic stenosis and valvular regurgitation, as well as inflammatory and genetic cardiomyopathies, are known to be associated with elevated levels of plasma vasoactive hormones. Although it is generally claimed that different vasoactive hormones such as catecholamines, angiotensin II, vasopressin, serotonin, and endothelins are involved in the pathogenesis of heart failure, the exact mechanisms for their involvement in the development of cardiac dysfunction in various diseases are not fully understood. Since heart failure is mostly preceded by adaptive cardiac hypertrophy, it is not clear how these vasoactive hormones participate in the transition of adaptive cardiac hypertrophy to maladaptive hypertrophy and progression to heart failure. In this article, we have, therefore, updated the existing information and described the evidence that these vasoactive hormones, through acting on their respective receptors, stimulate different prohypertrophic signal transduction mechanisms in cardiomyocytes for the induction of cardiac hypertrophy. The activation of receptors via different hormones has also been shown to stimulate sarcolemmal NOX2 for the production of oxyradicals and change the redox status of cardiomyocytes, which is considered to promote the hypertrophy process and the development of cardiac hypertrophy. In addition, these vasoactive hormones increase intraventricular pressure, ventricular wall tension and shear stress by inducing marked changes in the hemodynamic overload and inotropic effect on the myocardium. The vasoactive hormones also act on fibroblasts and promote the formation of collagen in the extracellular matrix as well as the development of apoptosis and replacement fibrosis for the occurrence of maladaptive cardiac hypertrophy by elevating the levels of proinflammatory cytokines such as IL-6 and TNF-α in cardiomyocytes. It is, thus, evident that initial events involved in the increase of cardiac muscle mass are associated with adaptive cardiac hypertrophy, whereas those dealing with the development of myocardial replacement fibrosis and the accumulation of collagen in the extracellular matrix are associated with maladaptive cardiac hypertrophy.

Although all vasoactive hormones are known to produce cardiac hypertrophy upon stimulating protein synthesis through their specific but complex receptor-mediated signal transduction mechanisms [31,32,33,34,35,36,37,38,39,40], the nature of proteins involved in the growth of cardiomyocytes, smooth muscle cells and fibroblasts seems to depend upon the type of hormonal stimulus as well as clinical stage and experimental models of cardiac remodeling and dysfunction. For example, YAP (yes-associated protein 1) has been demonstrated to activate the nuclear effector of the Hippo pathway by upregulating glucose transporter 1 (GLUT1), promoting glycolysis and inducing the accumulation of serine, aspartate and malate in physiological cardiac hypertrophy [264]. On the other hand, STING (stimulation of interferon gene) has been shown to induce pathological hypertrophy by upregulating inflammatory response and fibrosis upon increasing the expression of phospho-protein kinase RNA-like endoplasmic reticulum (ER) kinase and phospho-inositol-requiring kinase (all indices of ER stress) [265]. Furthermore, pathological cardiac hypertrophy was attenuated with HINT1 (histidine triad nucleotide-binding protein 1) by suppressing the expression of HOXA5 (homeobox A5) and inhibiting protein kinase Cβ type 1 and the MAP kinase/extracellular signal-regulated kinase/ yin yang 1 signal pathway [266]. From such complex observations, it can be appreciated that it is difficult to describe the exact nature of proteins involved in the development of cardiac hypertrophied and failing hearts. Particularly, it is pointed out that more than one vasoactive hormone is involved in initiating the hypertrophic process, which may not only exert their effects due to their own receptor-mediated signal transduction system but there may also occur cross talk between their receptor mechanisms. For example, Ang II has been shown to release catecholamines [267,268,269] and facilitate the formation of endothelin [270,271,272] and, thus, the influence of adrenoreceptor and endothelin receptor activations in the development of cardiac hypertrophy due to Ang II cannot be ruled out. Furthermore, Ang II is known to affect different isoforms of NOX, which may result in Na^+^ retention by activating the epithelial Na^+^-channels in the distal nephron, promoting Ca^2+^-influx in smooth muscles for increasing blood pressure and inducing inflammation for producing cardiac fibrosis [273,274,275]. Such indirect effects of Ang II on NOX isoforms with respect to hemodynamic overload and inflammation can also be seen to affect the Ang II-induced pathological cardiac hypertrophy due to its receptor-mediated signal transduction. In addition, it should be mentioned that the pro-hypertrophic, proinflammatory and pro-fibrotic actions of AT_1_R receptor activation through Ang II are antagonized via the activation of AT_2_R as well as MasR activation via Ang II metabolite, Ang1–7 [101,102,103,104]. Thus, any imbalance between the pro- and anti-inflammatory mediators leads to the transition of adaptive cardiac hypertrophy to maladaptive cardiac hypertrophy.

It needs to be emphasized that the vasoactive hormones are not only involved in the genesis of cardiac hypertrophy but are also considered to participate in the progression of cardiac maladaptive hypertrophy to heart failure. For example, both catecholamines and serotonin have been reported to enter cardiomyocytes and produce oxyradicals during their oxidation by mitochondrial monoamine oxidase. On the other hand, angiotensin II and endothelins generate oxyradicals by activating both sarcolemmal and mitochondrial NOX 4, whereas vasopressin may produce oxyradicals indirectly through mechanisms associated with the release of endogenous norepinephrine and angiotensin II due to increased intraventricular pressure and ventricular wall stress. The excessive generation of oxyradicals through diverse mechanisms such as the activation of xanthine oxidase and impaired electron transport in mitochondria can also be seen to develop oxidative stress in the hypertrophied heart. Furthermore, the depletion or depression in the antioxidant reserve in the hypertrophied heart via vasoactive hormones would favor the development of oxidative stress during the progression of heart failure. It should be mentioned that oxidative stress is known to activate metalloproteinases, produce the breakdown of collagen crosslinks and destabilize the extracellular matrix. In addition, oxidative stress has been reported to depress cardiac genes and activate calpain and other proteases either directly or indirectly through changes in the concentration of intracellular Ca^2+^; these alterations will induce subcellular defects and Ca^2+^-handling abnormalities in the hypertrophied heart. The increase in intracellular Ca^2+^ concentration in cardiomyocytes will also induce mitochondrial Ca^2+^-overload due to the action of different vasoactive hormones. This change will not only impair the process of energy production but will also generate oxyradicals in the myocardium. Thus, oxidative stress may result in the development of cardiac dysfunction and play an important role in the progression of cardiac hypertrophy to heart failure due to vasoactive hormones. It is also pointed out that the failure of several classical treatments such as inhibitors of the RAS and β-adrenoreceptor blockers to improve the long-term outcome of heart failure is due to the fact that such interventions were developed to suppress the effects of a single vasoactive hormone. Accordingly, in view of the involvement of several vasoactive hormones in the development of heart failure, it is suggested that some special combination therapy using different receptor antagonists be designed to improve the treatment of this devastating health hazard.
